# Study of diffuse phase transition and relaxor ferroelectric behavior of Ba_0.97_Bi_0.02_Ti_0.9_Zr_0.05_Nb_0.04_O_3_ ceramic

**DOI:** 10.1039/c8ra08910h

**Published:** 2019-01-18

**Authors:** Z. Raddaoui, S. El Kossi, J. Dhahri, N. Abdelmoula, K. Taibi

**Affiliations:** Laboratoire de la Matière Condensée et des Nanosciences, Université de Monastir, Faculté des Sciences de Monastir Avenue de l'environnement 5019 Monastir Tunisia j.dhahri3000@gmail.com; Laboratoire des Matériaux Ferroélectriques, LR-Physique-Mathématiques et Applications, Université de Sfax, Faculté des Sciences Route de Soukra km 3.5 B.P 1171 3000 Sfax Tunisia; Laboratoire de Sciences et Génie des Matériaux, Faculté de Génie Mécanique et Génie des Procédés, Université des Sciences et de la Technologie Houari Boumediene BP32 Bab Ezzouar 16111 Alger Algeria

## Abstract

In the present work, structural and dielectrics properties of polycrystalline sample Ba_0.97_Bi_0.02_Ti_0.9_Zr_0.05_Nb_0.04_O_3_ (BBTZN) prepared by a molten-salt method were investigated. X-ray diffraction analyses revealed the formation of a single-phase pseudocubic structure with a *Pm*3̄*m* space group. Unlike the trend observed in classic ferroelectrics, the temperature dependence of the dielectric constants showed the presence of three sequences of structural phase transitions. In fact, the local disorder provides a frequency dependent relaxor like behaviours attributed to the dynamic of polar nanoregions (PNRs). The diffuse phase transition (DPT) analyzed using the modified Curie–Weiss law and Lorenz formula confirms the presence of short-range association between the nanopolar domains. The obtained values of the degree of diffuseness are found to be in the range of 1.58–1.78 due to the existence of different states of polarization and, hence, different relaxation times in different regions. The frequency dependence of temperature at dielectric maxima, which is governed by the production of PNRs at a high temperature, satisfies the Vogel–Fulcher (V–F) law. The temperature dependence of the electric modulus for various frequencies indicating a thermally activated relaxation ascribed to the Maxwell–Wagner (M–W) space charge relaxation phenomenon.

## Abstract 2

In the present work, structural property of polycrystalline sample Ba_0.97_Bi_0.02_Ti_0.9_Zr_0.05_Nb_0.04_O_3_ (BBTZN) prepared by a molten-salt method were investigated.

## Graphical abstract

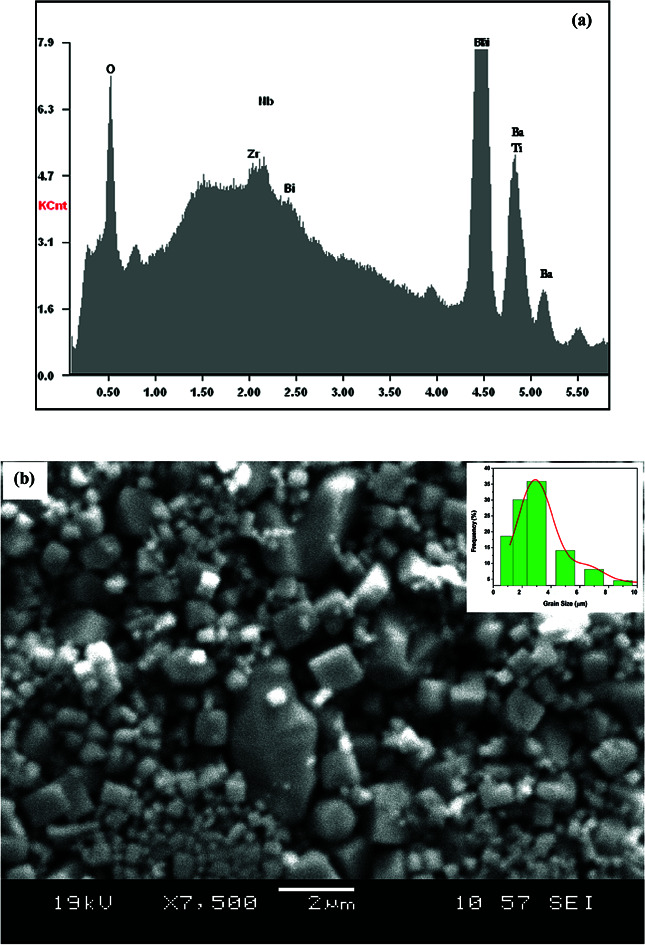

## 1. Introduction

New industrial applications require advanced materials with interesting properties. Perovskite materials fall into this category of materials fall into this category of materials owing to their promising physical properties and potential manipulation in many industrial and engineering fields such as multilayer ceramic capacitors MLCCs, positive-temperature-coefficient resistors PTCRs, and piezoelectric transducers.^1–3^ However, these days much research is directed towards lead free relaxor materials with a perovskite structure characterized by a translational symmetry breaking to form polar nanoregions (PNRs), which explains the origin of relaxor behavior.^4^ In this respect, compositional inhomogeneity plays a central role, and discrete ferroelectric nano-regions have been proposed to explain the diffused dielectric response.^5^ The first evidence of these inhomogeneities was provided by Burns and Dacol^6^ who observed a deviation from the linear temperature dependence of the optical index of refraction below a certain temperature during the cooling of the La-doped PbZr_(1−*x*)_Ti_x_O_3_ samples.

Despite, the discovery of many attractive properties in some perovskite structures including relaxor behavior in Pb(Mg_1/3_Nb_2/3_)O_3_–PbTiO_3_,^7,8^ Pb(Mg_1/3_Nb_2/3_)O_3_ Pb(Zr_0.52_Ti_0.48_)O_3_,^9^ and Pb(Zn_1/3_Nb_2/3_)O_3_–PbTiO_3_,^10^ they have net been put to application due to their volatility and toxicity. So many researchers have attempted to overcome this problem by doping BaTiO_3_ in the A or/and B sites to become a relaxor.^11^ In fact, the discovery of the high-permittivity of the ferroelectric ceramic BT in 1943 (ref. 12) mode it subject of investigation in order to understand and manipulate its properties. These compounds present several polymorphic phase transitions used to correlate important dielectric/ferroelectric properties, such as variations in polymorphic phase transition temperatures, permittivity, and dielectric loss with chemical doping and ceramic microstructure.^13–15^

Incorporating Bi^3+^ in to this structure at Ba site was reported in many works and assumed that the relaxor behavior is induced by heterovalent substitution can be explained by the random-field-induced domain state.^16–19^

Moreover, other researchers studied the effect of doping at Ti^4+^ site using more stable chemical ions and larger ionic size as Zr^4+^. They report a decrease of the electronic hopping conduction, and the existence of introduce a relaxor behavior in the BT attributed to the distribution of micro-polar regions along the structure.^20^ Introducing disorder on BT using Nb^5+^ lead to small inhomogeneities distribution and the creation of titanium vacancies.^21^ It is evident that more efforts are needed in this area in order to produce an ideal, low loss, highdielectric constant material that could be used for advanced capacitive applications.^22^

Therefore, in this work we have synthesized the Ba_0.97_Bi_0.02_Ti_0.9_Zr_0.05_Nb_0.04_O_3_ (BBTZN) ceramic by the molten-salt method. We have studied the effect of structure and microstructure on the dielectric properties of the polycrystalline BBTZN.

## 2. Experimental details

### 2.1. Preparation of Ba_0.97_Bi_0.02_Ti_0.9_Zr_0.05_Nb_0.04_O_3_ perovskite sample

A polycrystalline BBTZN sample was produced by the molten salt method using stoichiometric amounts of BaCO_3_, Bi_2_O_3_, TiO_2_, ZrO_2_ and Nb_2_O_5_ (having a purity of more than 99.9% for each of them). The precursors were weighed, then, thoroughly mixed in an agate mortar for 2 h. The salt-precursor mixture was placed in an alumina crucible and heated at 800 °C for 24 h. After cooling to room temperature, the mixture was washed with distilled water and filtered to remove the salts. After being dried at 100 °C in air and ground thoroughly, the compounds were pressed into disks (with a diameter of 8 mm and a thickness about 2 mm), and sintered in air at 900 °C for 24 h.

### 2.2. Characterization of the sample

The composition and microstructure of the BBTZN ceramic were analysed using scanning electron microscopy (SEM) equipped with an energy dispersive X-ray system (EDX). The pictures were taken at room temperature on a Phillips XL30 microscopy.

The crystal structure, as well as its purity were checked by X-ray diffraction (XRD) using “PANalytical X'Pert Pro” diffractometer with CuK_α_ radiation. Data a were collected at room temperature in the range of 2*θ* from 10 to 70° with a step size of 0.017° and a counting time of 18 s per step. The structural analysis was carried out by the standard Rietveld method.^23^

The dielectric properties were measured on discs under helium atmosphere as a function of both temperature (290–800 K) and frequency (1 kHz to 1 MHz) using a Wayne-Kerr 6425 component analyzer. All the dielectric data were collected while heating at a rate of 2 K min^−1^.

## 3. Results and discussion

**Fig. 1:**
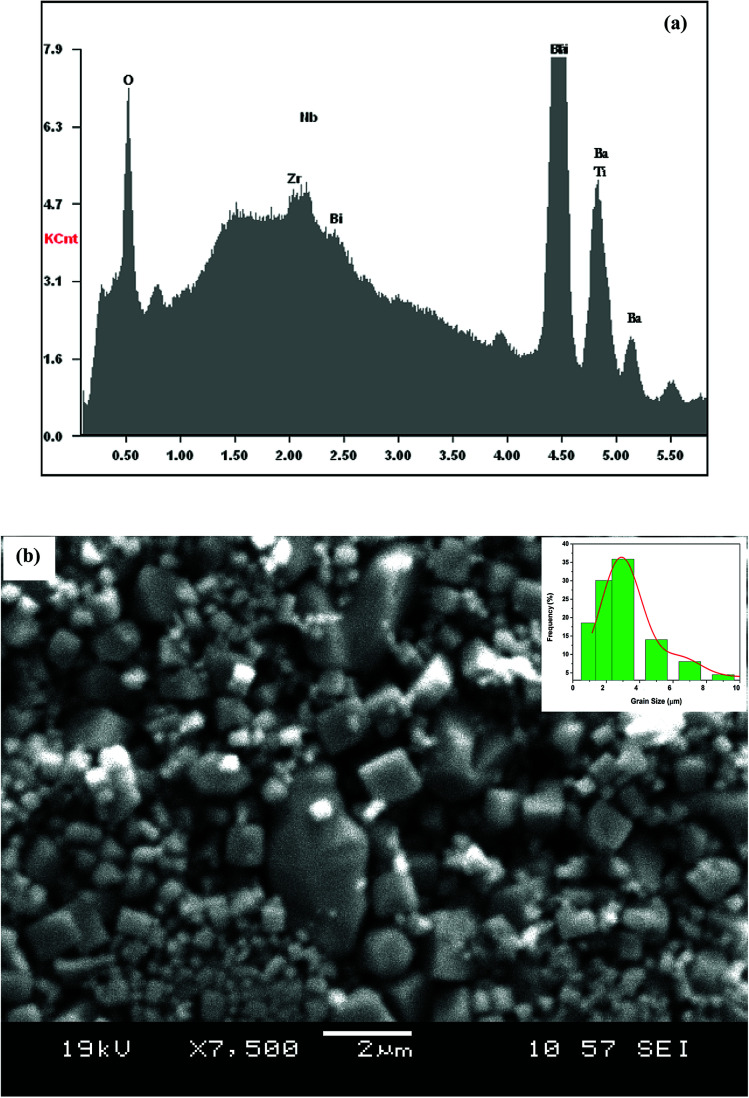
(a) EDX analysis spectrum of Ba_0.97_Bi_0.02_Ti_0.9_Zr_0.05_Nb_0.04_O_3_ ceramic; (b) SEM image of the sample. The inset shows the size distribution histogram.

The EDX chemical analysis of Ba_0.97_Bi_0.02_Ti_0.9_Zr_0.05_Nb_0.04_O_3_ sample is shown in Fig. 1(a). The EDX spectrum reveals the presence of Ba, Bi, Ti, Zr, Nb and O elements, which confirms that there is no loss of any integrated element during sintering, within the experimental errors. EDX analysis also showed that the chemical composition of the sample is close to the nominal one within the experimental uncertainties.

The surface of the BBTZN sample is shown in Fig. 1(b). It can be seen from the micrograph that the inhomogeneous microstructures of the grains and may be advantageous to explain the dielectric properties.^24^

The size distribution of particles presented in the inset of Fig. 1(b) was analysed quantitatively by fitting the histogram using a Lorentzian function. The mean diameter of BBTZN is of 2.5 μm order.

**Fig. 2:**
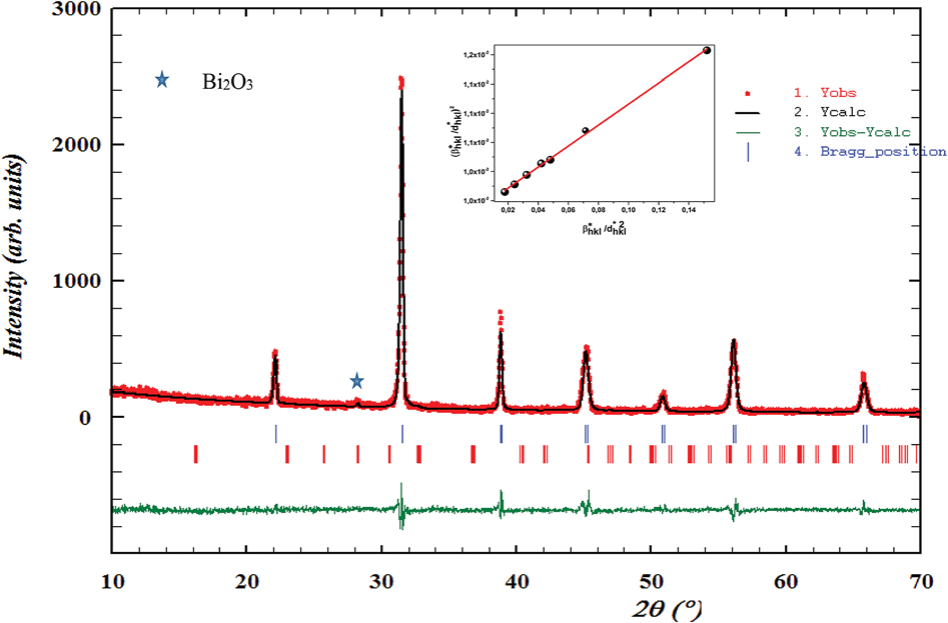
Room temperature XRD pattern and Rietveld refinement result for Ba_0.97_Bi_0.02_Ti_0.9_Zr_0.05_Nb_0.04_O_3_ ceramic. The inset shows their crystal structure and fitted curves of the H–W analysis.

The X-ray diffraction patterns for the polycrystalline BBTZN sample taken at room temperature are presented in Fig. 2. This figure, shows that the sample is in a single-phase condition with minor secondary phases identified as Bi_2_O_3_ phase arising from a small contamination during the sample preparation, which is in agreement with the results found by A. Aoujgal *et al.*^25^ The concentration of this impurity is obviously very small (a few percent). The structural parameters, unit cell volume and fitting parameters of the sample were refined by the standard refinement analysis. This refinement was performed with *Pm*3̄*m* space group in the pseudocubic unit cell. The related results are listed in Table 1.

**Table:** Refined structure parameters for Ba_0.97_Bi_0.02_Ti_0.9_Zr_0.05_Nb_0.04_O_3_ after the Rietveld refinement of X-ray diffraction data at room temperature

	Ba_0.97_Bi_0.02_Ti_0.9_Zr_0.05_Nb_0.04_O_3_	
	Structure pseudo-cubic	*Pm*3̄*m* phase
Cell parameters	*a* = *b* (Å)	4.0135(4)
	*V* (Å^3^)	64.652(1)
Thermal agitation	(Ba/Bi) *B*_iso_ (Å^2^)	1.488
	(Ti/Zr/Nb) *B*_iso_ (Å^2^)	1.731
	(O_1_) *B*_iso_ (Å^2^)	2.002
Bond lengths and Bond angles	*d*_Ti–O_ (Å)	2.006(7)
	*θ*_(Ti–O–Ti)_ (°)	180.00
Discrepancy factors	*R*_p_ (%)	9.57
	*R*_wp_ (%)	12.5
	χ^2^	1.78
Experimental density	*d*_theo_ (g cm^−3^)	6.142
Theoretical density	*d*_exp_ (g cm^−3^)	5.97
Compactness	*C*	0.97
Average crystallite size	〈*D*〉_XRD_ (nm)	64.2
	〈*D*〉_H–W_ (nm)	91
	*ε*	0.002

XRD profile analysis is a simple and powerful method to determine the average crystallite size D_XRD_. Debye–Scherrer's formula showed the broadening of the XRD pattern attributed to the crystallite size-induced broadening:^26^1
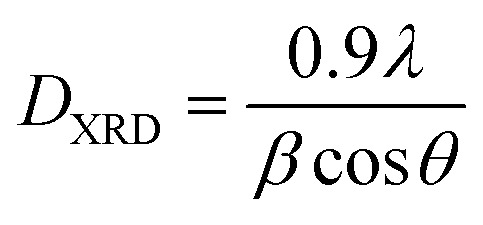
where *λ* is the used X-ray wavelength, *θ* and *β* are, respectively, the diffraction angle and the full width at half-maximum of the most intense peak. The value of the average crystallite size (*D*_XRD_) is listed in Table 1.

The crystallite size calculated by XRD data (*D*_XRD_) was smaller than that obtained by SEM image, which indicates that each particle observed by SEM is formed by several crystallized grains.^27^

According to the Halder–Wagner (H–W) method, the crystallite size (*D*_H–W_) and the strain (*ε*) of the powder can be determined using the *β*_*hkl*_ and planar spacing *d*_*hkl*_ (the distance between adjacent planes in the set (*hkl*)):^28^2
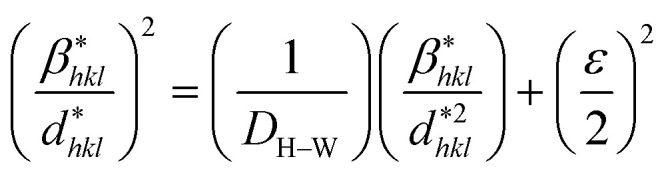
where 
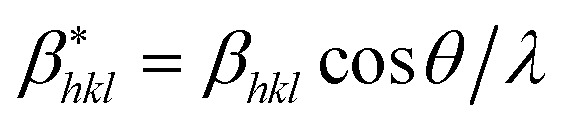
 and 
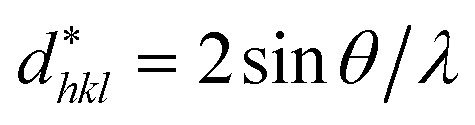
. The results of crystallite size and micro-strain are summarized in Table 1. The plot of eqn (2) is a straight line with a positive slope and a nonzero *y*-intercept (the inset of Fig. 2). The crystallite size *D*_H–W_ was determined from the slope inverse of the linearly fitted data and the root of the *y*-intercept gave the microstrain. The value of the crystallite size (*D*_H–W_) obtained from the H–W analysis was compared to that obtained by Scherrer's method (*D*_XRD_).

**Fig. 3:**
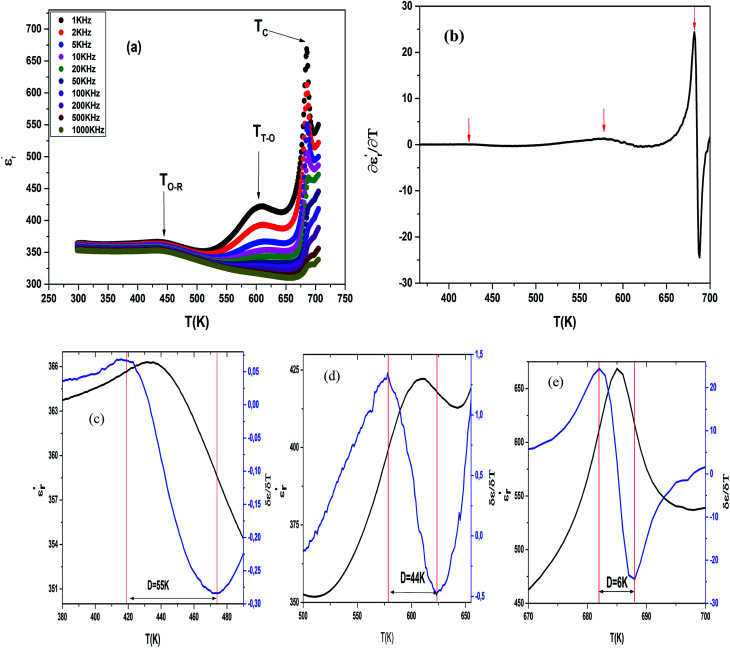
Temperature dependence of dielectric properties of Ba_0.97_Bi_0.02_Ti_0.9_Zr_0.05_Nb_0.04_O_3_ at different frequencies (a), variation of 
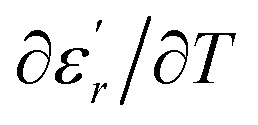
 of Ba_0.97_Bi_0.02_Ti_0.9_Zr_0.05_Nb_0.04_O_3_ ceramic (b), the value of the diffuseness degree for this composition of region I (c), region II (d), region III (e).

The temperature and frequency variations of the dielectric constant of BBTZN ceramics are shown in Fig. 3(a). To highlight the phase transitions, we plotted the curve of the differential dielectric constant 
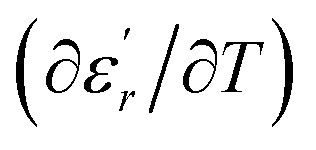
*vs.* temperature in the insets of Fig. 3(b). Three peaks were observed on the dielectric curves. They originated from phase transitions from a cubic paraelectric to a tetragonal ferroelectric at the Curie temperature (*T*_C_), then to an orthorhombic ferroelectric (at *T*_T-O_), and finally to a rhombohedral ferroelectric (at *T*_O-R_). These transitions are identical than those obtained on pure BT ceramics.^29^ Some researchers have reported low values of dielectric constant at *T*_c_ such that Li *et al.*^30^ report the maximum dielectric constant 
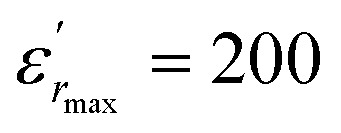
, Hayashi *et al.*^31^ is of the order 
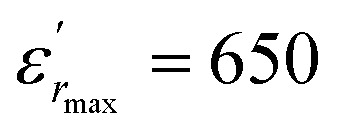
.

Three regions of dielectric relaxations were observed in the present perovskite at the temperature ranges of 350–500 K, 500–620 K and 620–720 K with a maximum in the dielectric permittivity that shifted to a higher temperature with increasing frequency (Fig. 3(a)). This shift of each maximum in the dielectric permittivity for the three regions indicating diffused type phase transition. All the observed dielectric behaviours confirm the diffuse relaxor character for our composition. This indicates an important property of a disordered perovskite structure. In addition, we notice coexistence of low dielectric constant and high dielectric constant regions in dielectric materials. It was explained by the charge accumulation at the interface between the grain and grain boundaries (as shown in SEM images (Fig. 1(b))) and lead to a Maxwell–Wagner (MW) interfacial polarization.^32^

Compared to literature 33–35 (Table 2), we note that a low dielectric constant of the BBTZN sample, attribute able to the presence of Bi, Zr, Nb in the BaTiO_3_ lattice, which can minimize the local constraints and facilitate the motions of the domain. This great mobility of the domain walls in this ceramic, led to a low dielectric constant.

**Table:** Parameters obtained from the temperature dependence of dielectric permittivity

Compounds	Ref.	*ε*_m_ (1 kHz) at *T*_c_	*T*_c_
Ba_0.97_Bi_0.02_Ti_0.9_Zr_0.05_Nb_0.04_O	This work	669	655
BaTiO_3_	29	200	415
BaTiO_3_	30	650	—

The broad peak or diffusiveness in *ε*′(*T*) occurred due to fluctuations in the composition and structural disorder in the cationic arrangement on one or more site of the crystal structure. The local disorder in the system led to the formation of polar nanoregions (PNRs) of different sizes and local curie points. The dynamics of these PNRs is responsible for the diffuse phase transition (DPT).^36^

The width of the transition represents the DPT, which is very important in ferroelectric materials.

We can define ‘*D*’ is the characterizing parameter of diffuseness degree. It is the temperature interval where the volume of polar microscopic regions changes due to the appearance of new microscopic polar regions. This is defined as:^37^3
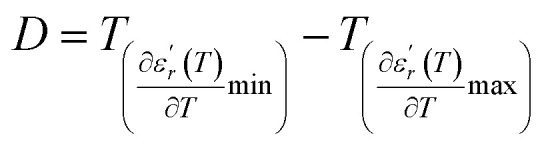
where 
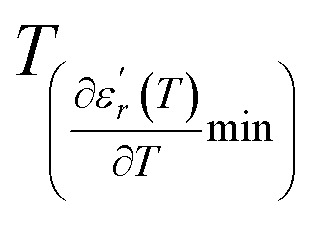
 and 
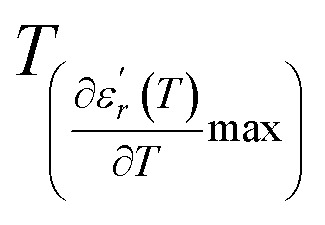
 are the temperature when 
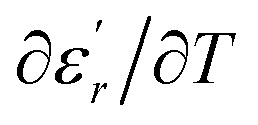
 reaches the minimum and maximum, respectively. The curves for the 
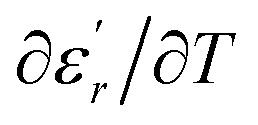
 against (*T*) for the three regions I, II and III are shown in Fig. 3(c–e). The temperature interval between 
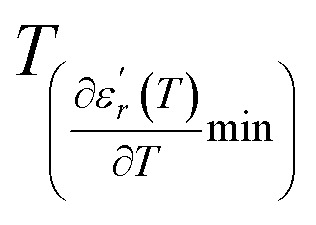
 and 
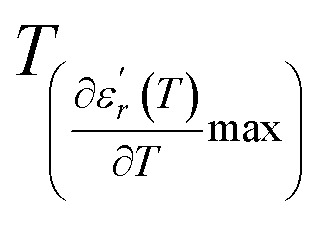
 reflects the diffuseness degree microscopically. The vertical dashed lines in Fig. 3(c–e) correspond to 
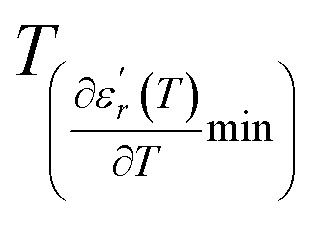
 and 
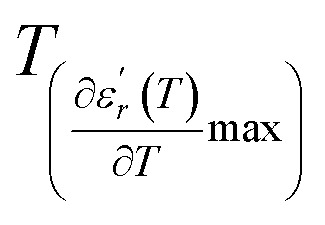
 values. *D* is the interval between 
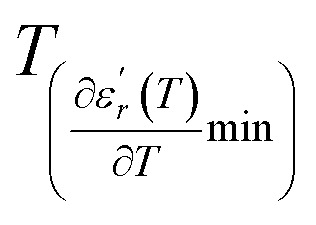
 and 
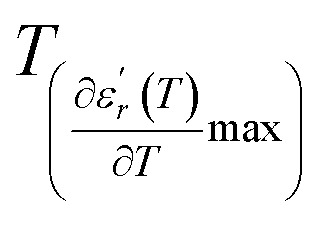
.

The values of diffuseness decreases with the increase in temperature. This indicates that the phase transition from paraelectric-ferroelectric is the transition that occurs with less diffuse (*D* = 6 K) disorder, while the phase transition tetragonal to orthorhombic phase transition is the transition that occurs with more diffuse (*D* = 44 K) disorder. The orthorhombic to rhombohedral phase transition is the transition more diffuse (*D* = 55 K) disorder.

To explain the ferroelectric behavior with DPT phenomenon of the dielectric materials, Curie and Weiss propose the Curie–Weiss law.^38^ When the temperature is above *T*_c_, the dielectric normal behavior follows the C–W law as follows:4
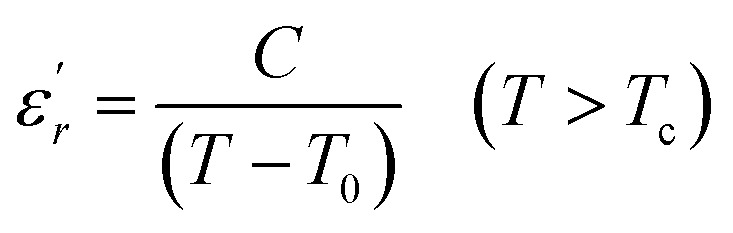
where *C* and *T*_0_ are the C–W constant and the C–W temperature, respectively.

**Fig. 4:**
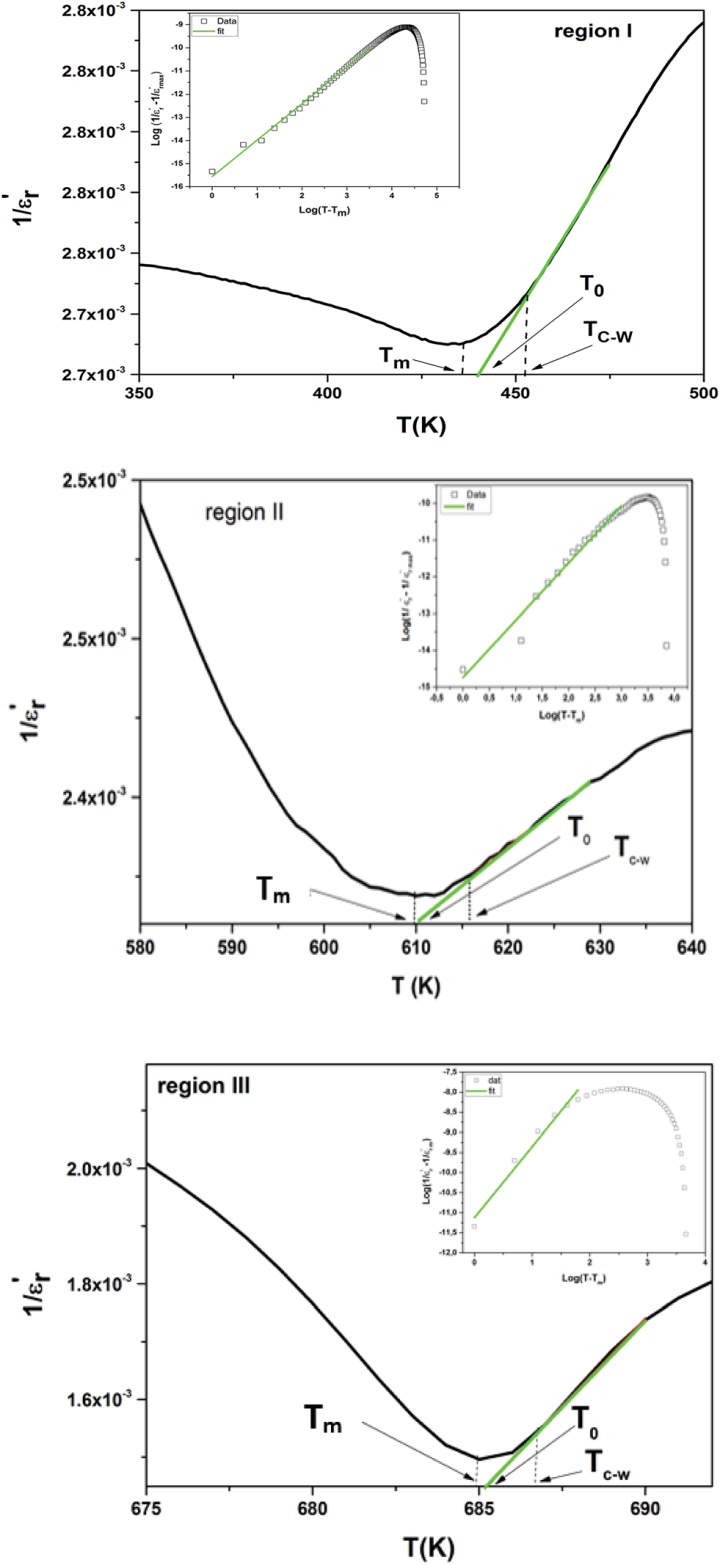
Temperature dependence of inverse of permittivity 1/*ε*′ at 1 kHz for Ba_0.97_Bi_0.02_Ti_0.9_Zr_0.05_Nb_0.04_O_3_ ceramic: region I, region II and region III. The insets are plots of 
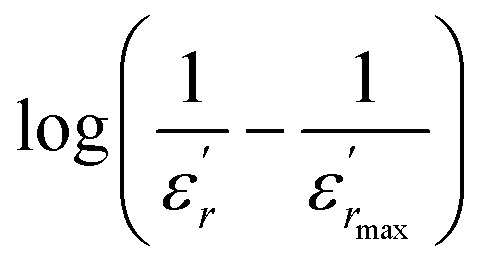
 as a function of log(*T* − *T*_m_) at 1 kHz for the compound: region I, region II and region III.

The inverse of dielectric constant 
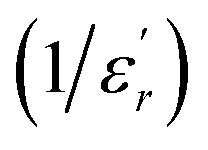
 as a function of temperature at 1 kHz for BBTZN ceramic sample is plotted in Fig. 4. The two parameters *C* and *T*_0_ obtained by fitting the inverse dielectric to eqn (4) are summarized in Table 2.

The value of Curie constant obtained is in the order of 10^5^ that is normally observed for displacive ferroelectrics type. (*T*_C_ ≠ *T*_0_) shows that the phase transition is of a first order type.^39^ All these dielectric results are in agreement with relaxor ferroelectric behavior for three regions.

In addition, we notice that the curves can be divided into three regions. The first region represents the ferroelectric behavior up to the transition at *T*_m_. The second region, near the transition, indicates a diffused transition up to a temperature *T*_C–W_. The third region represents the linear behavior of 1/*ε*′ as a function of temperature following the Curie–Weiss relation. It is clear from the above figures that for both samples at *T* > *T*_C–W_, a normal Curie–Weiss behavior is found.

The parameter Δ*T*_m_, which is often used to show the degree of deviation from the Curie–Weiss law, is defined as follows:

5 Δ*T*_m_ = *T*_C–W_ − *T*_m_

where *T*_m_ denotes the temperature of the dielectric constant maximum 
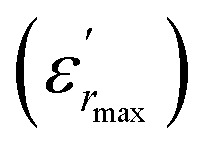
 and *T*_C–W_ represents the temperature from which the dielectric constant starts to deviate from the Curie–Weiss law. The various constants are listed in Table 2. The deviation from the Curie–Weiss behavior was qualified to a short-range association between the nanopolar domains. In addition, the deviation from Curie–Weiss law within a certain temperature interval above *T*_m_ is one of the salient features of relaxor ferroelectrics.

The relaxor behavior in ferroelectric compound is usually affected by the compositional fluctuation, the merging of micropolar regions into macropolar regions, or a coupling of order parameter and local disorder mode through the local strain.^40^

Because the radius of Bi^3+^ (0.117 nm) is smaller than that of Ba^2+^ (0.161 nm) and Zr^4+^ (0.072 nm), and the radius of Ti^4+^ (0.061 nm) is close to that of Nb^5+^ (0.064 nm),^41^ the complex substitution at A-site and B-site tends to cause inhomogeneous composition distribution and disordered crystal structure.

Therefore, according to our results, the observed relaxation phenomena is attributed to the multi-ion coexistence at the A and B-sites. Different effects of A-site substitution on cation ordering and the stability of the polar region are considered to be based on the polarizability of cations and the tolerance factor of the perovskite structure. For perovskites with the ABO_3_ general formula, the following equation can be used to calculate the tolerance factor (*t*):6
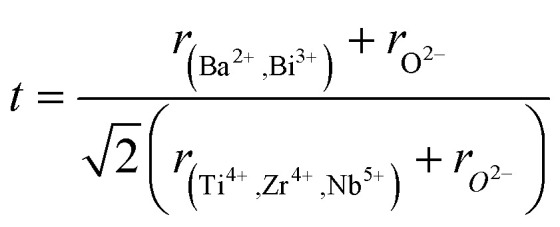


The tolerance factor value was estimated *t* = 0.93. It was found within the range of a stable perovskite structure.

So, Ba^2+^ cations can stabilize normal ferroelectrics due to the larger ionic diameter and higher polarizability. While Bi^3+^ cations in A-sites behave as a typical destabilizer against normal ferroelectrics and induce paraelectric behavior due to smaller ionic diameter and lower polarization. In this case more macrodomains (long-range ordered regions) in Bi substituted ceramic will breakup into micropolar regions than those in BaTi_0.9_Zr_0.05_Nb_0.04_O_3_ ceramic. Mechanical stress in the grain is one of the causes of relaxor behavior in the Ti^4+^, Zr^4+^ and Nb^5+^ mixed composition.

Similar behaviors are reported for Ba_(1−*x*)_Bi_2*x*/3_Zr_0.15_Ti_0.85_O_3_ and was attributed to the diffuse ferroelectric phase transition.^42^ N. Haddadou *et al.*^43^ report for the Ba_0.925_Bi_0.05_(Ti_0.95−*x*_Zr_*x*_)Sn_0.05_O_3_ (0.05 ≤ *x*≤ 0.30) ceramics than the low Zr-composition was found to exhibit a flat 
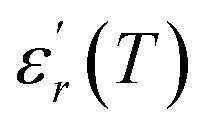
 curve with stable dielectric permittivity in a large range of temperature, promising for the X7R specifications.

An empirical relaxation strength characterizes the frequency dispersion and could be defined by the parameter Δ*T*_relax_ = *T*_m_ (1 MHz) − *T*_m_ (1 kHz).^44^ The values of Δ*T*_relax_, as shown in Table 2, the values of Δ*T*_relax_ are higher in the regions (I and III). The substitution of Bi^3+^ for Ba^2+^ in site A promote the relaxor behavior with a low amplitude of dielectric constant.^45^ Also substituting Nb^5+^, Zr^4+^ for Ti^4+^ to heightens the transition temperatures. According to the bibliography for rate of ion Zr^4+^ = 5% we found the diffuse phase transition^46,47^ and the effect of the Nb^5+^ ions to improve the dielectric property.^48^

To further study the phase transition behavior of the BBTZN sample, we calculated the diffuseness parameter using the modified Curie–Weiss law:^49^7
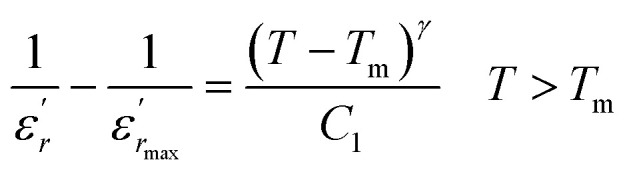


The diffuseness coefficient *γ* allows understanding the character of the phase transition; *C*_1_ is a constant quantity and 
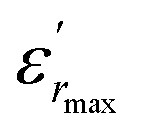
 is the peak dielectric permittivity at temperature *T*_m_. In general, the value of *γ* between these limits (1 <*γ* < 2) provide an incomplete diffuse phase transition. For normal ferroelectrics *γ* approximate to 1 but relaxor ones approximate to 2.

The inset of Fig. 4 shows the plots of 
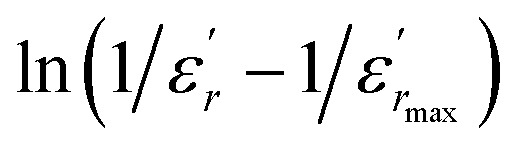
*versus* ln(*T* − *T*_m_) at 1 kHz for BBTZN ceramic sample. The obtained values of ‘*γ*’ are found to be in the range of 1.58–1.78 (Table 2), which corresponds to the diffuse phase transition due to the existence of different states of polarization and, hence, different relaxation times in different regions.^50^

The values of ‘*γ*’ indicate that the paraelectric-ferroelectric phase transition (*γ* = 1.78) is the transition that occurs with more disorder, while the phase transition from orthorhombic to rhombohedral (*γ* = 1.58) is the transition that occurs with less disorder.

The incorporation of Bi_2_O_3_ induced the ferroelectric relaxor behavior in the Ba_0.8_Sr_0.2_TiO_3_ perovskite. The relaxor character based on empirical parameters such as *γ* and the originated is form the micro-polar clusters, which stemmed from the heterovalent substitutions of Bi^3+^ at A site.^42^

In addition, the relaxor behavior with DPT occurs at the B site of the ABO_3_ perovskite structure, where Nb^5+^ and Ti^4+^ also possess different valences and ionic radii, as reported by Z. Sun *et al.*^51^ for the perovskite Ba(Zr_0.2_Ti_0.8_)_(1−*x*)_Nb_*x*_O_3_ ceramics. This difference for ionic radii produce the formation of the local electric fields owing to the local charge imbalance and the local elastic fields due to local structure distortions, prevents the long-range dipole alignment, *i.e.*, giving rise to the PNRs.

Therefore, the origin of the observed relaxor character in BBTZN is the substitution of Bi^3+^ for Ba^2+^ in A site and (Zr^4+^, Nb^5+^) for Ti^4+^ in B site. This inhomogeneous distribution leads to the compositional fluctuation which may result in microscopic heterogeneity with different Curie points and cause the observed diffuseness.^52^ Furthermore, it creates the different states of polarization and hence different relaxation time in different regions and also to the formation of a local charge imbalance and defects, which cause, in turn, the thermal activation conduction of mobile ions/or the other defects contributing to the observed dielectric dispersion.^53^ However, many studies have shown that *γ* is not a good parameter to give the exact degree of dielectric relaxation in perovskites.^54^ Recently, both empirical relations, Gaussian-distribution above *T*_m_ and the better Lorentz type above and below *T*_m,_ have been attempted to express fairly the relaxor feature. Smolenskii and co-works^55^ introduced the concept of Gaussian distribution of the *T*_m_ to the small micro-regions considered noncorrelated. According to such an assumption, the permittivity *ε*_*r*_ as a function of temperature is expressed as:^56^8
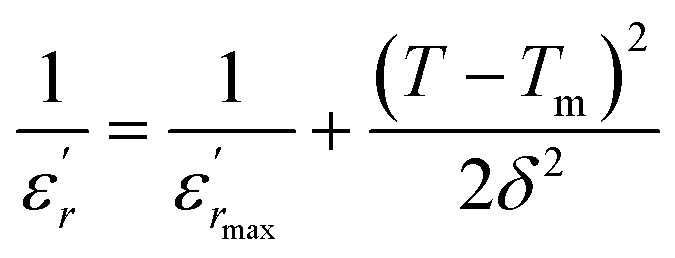
where 
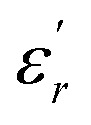
 is the dielectric constant, 
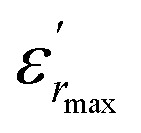
 is its maximum value, *T*_m_ is the temperature corresponding to this maximum, and *δ* is the Gaussian coefficient of diffuseness.

**Fig. 5:**
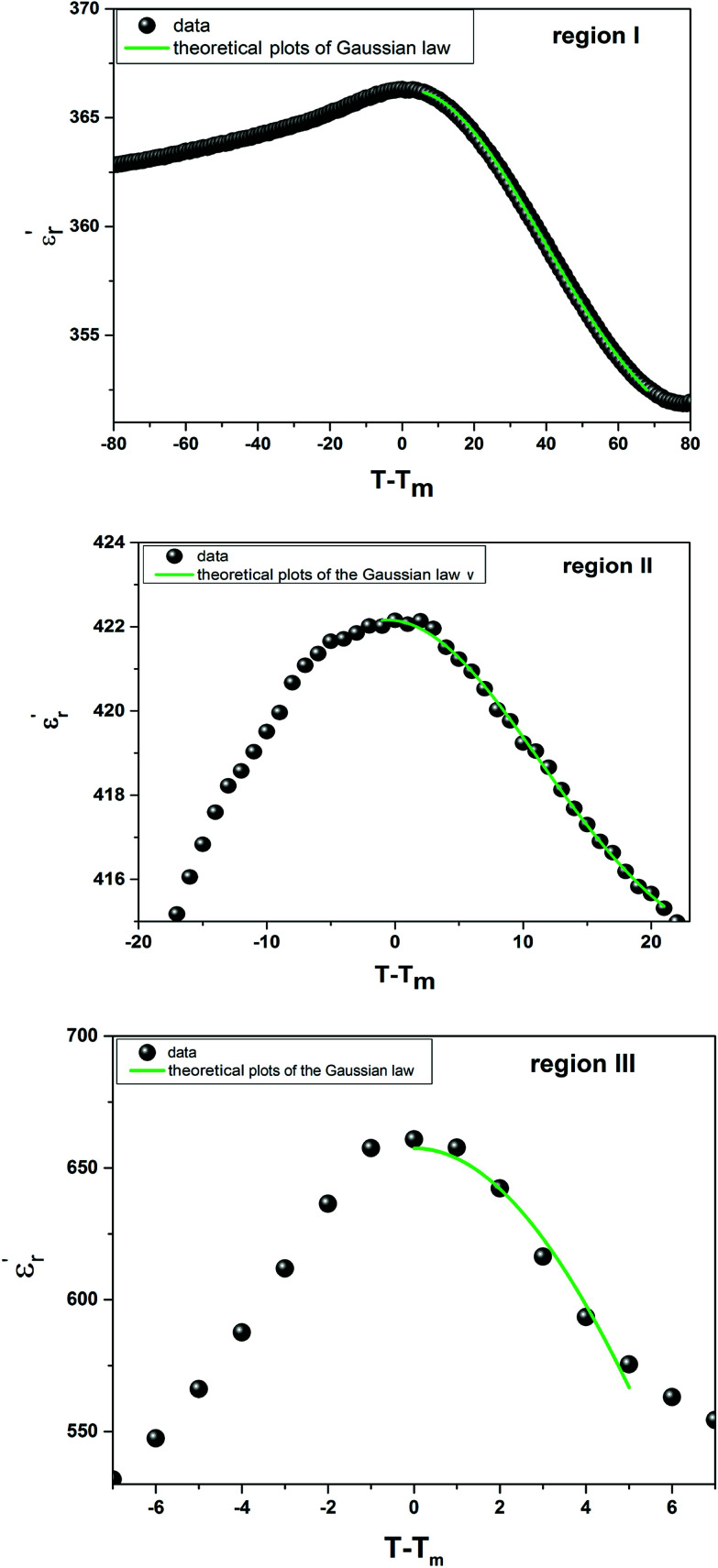
The experimental and fitted dielectric data as a function of temperature for Ba_0.97_Bi_0.02_Ti_0.9_Zr_0.05_Nb_0.04_O_3_ ceramic using the Gaussian distribution.

Fig. 5 shows the fitted results from eqn (8). The best Gaussian fitting parameter (*δ*) is shown in Table 3.

Gaussian distribution and Lorentz type fitting parameters of Ba_0.97_Bi_0.02_Ti_0.9_Zr_0.05_Nb_0.04_O_3_ ceramic

**Table:** 

	Δ*T*_relax_ (K)	Δ*T*_diffuse_ (K)	Δ*T*_m_ (K)	*C* (10^5^K)	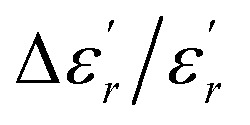	*γ*	*T*_m_	*ε*_m_ (1 kHz)	*T*_transitions_ (K)
Regions I	2	68	17	5.1	0.04	1.58	435	366	435
Regions II	11	90	8	0.2	0.92	1.6	612	422	612
Regions III	21	73	3	0.14	0.51	1.78	655	669	655

**Table:** 

Dielectric fitting parameters			Ba_0.97_Bi_0.02_Ti_0.9_Zr_0.05_Nb_0._3		
			Regions I	Regions II	Regions III
Gaussian distribution	*T* < *T*_m_	*δ* (K)	35.9	113.48	9.17
		χ^2^	0.99	0.95	0.97
Lorenz type	*T* < *T*_m_	*ε*_A_	366.5	430	695
		*T*_A_ (K)	448.39	628.35	689.69
		*δ*_A_ (K)	443.4	126	25
		χ^2^	0.98	0.997	0.986
	*T* > *T*_m_	*ε*_A_	366.22	438.21	747
		*T*_A_ (K)	433.19	516.08	677.1
		*δ*_A_ (K)	182.96	346	16.86
		χ^2^	0.99	0.995	0.984

**Fig. 6:**
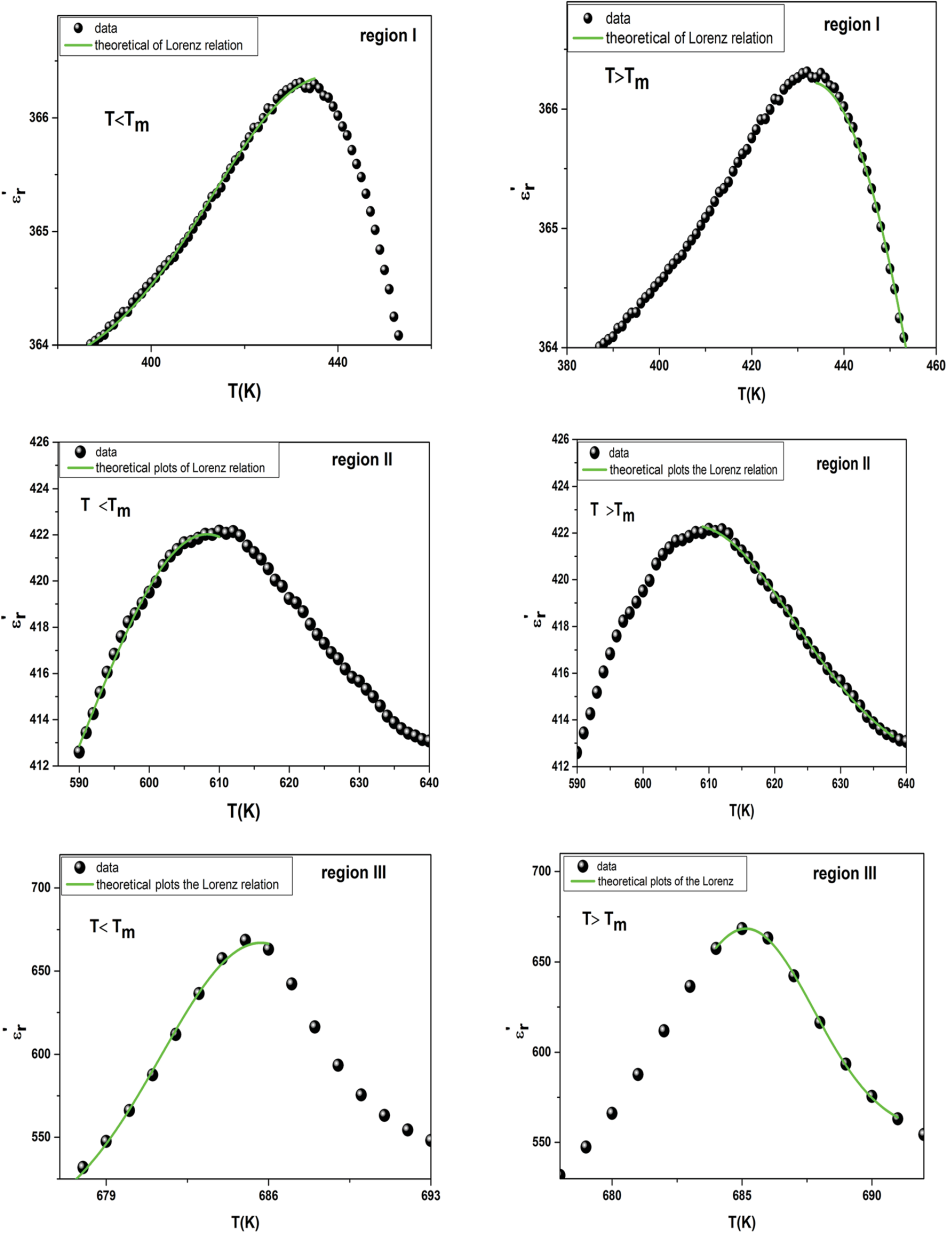
The experimental and fitted dielectric data as a function of temperature for Ba_0.97_Bi_0.02_Ti_0.9_Zr_0.05_Nb_0.04_O_3_ ceramic using the Lorenz type.

It has been emphasized in the literature that a description of the high temperature slope of the dielectric peak in ferroelectrics with or without DPT, and classical relaxor materials can be well described above and below *T*_m_ with the Lorenz formula empirical relation:^57^9
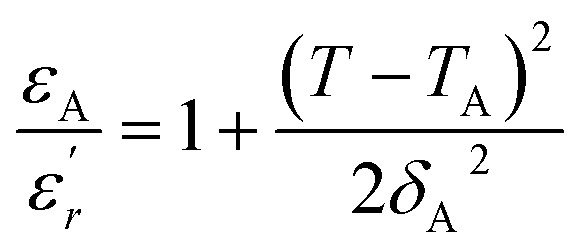
where *δ*_A_ reflects the diffuseness of the dielectric relaxation, *T*_A_ (*T*_A_ ≠ *T*_m_) and *ε*_A_ are the fitting parameters defining the temperature of the peak position and magnitude of the Lorentz peak. The temperature dependent dielectric data 
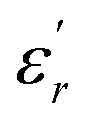
 measured on BBTZN ceramic could be fitted by the Lorentz-type quadratic relation in the two different temperature ranges *T* < *T*_m_ and *T* > *T*_m_, respectively. The values of *δ*_A_, *T*_A_ and *ε*_A_ obtained from the experimental data, fitted by eqn (9) are listed in Table 3, and the fitting curves are shown in Fig. 6.

We notice the very different fitting parameters *δ*_A_ in two different temperature ranges (*T* < *T*_m_ and *T* > *T*_m_), which indicates that there should be two polarization processes in these compound for the three transitions. Furthermore, *δ*_A_ increased in the two ranges of temperature, which suggests increasing the diffuseness. This behavior explains by the Bi^3+^ substitution in A site and (Zr^4+^, Nb^5+^) for Ti^4+^ in B site is found to induce chemical and structural inhomogeneity that leads to structural disorder reflected as increasing diffuseness in the transition.

Comparing the Lorenz type and Gaussian distribution for *T* > *T*_m_, we note that the diffuseness of the dielectric relaxation are close. From the temperature dependence of 
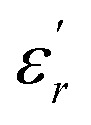
 for the compound BBTZN and the fitting process, it is noted that the Lorenz formula is more accurate than the Gaussian formula for description the ferroelectric phase transition. It is deduced that the Curie–Weiss law which describes the temperature dependence of the dielectrics in the three phase transitions. The difference of eqn (8) and (9) is that the former supposes Gaussian distribution of PNRs at *T*_C_, and the latter supposes Lorentz-type distribution. From the above analysis, eqn (9) could describe the experience phenomenon precisely.

In addition, the diffuseness of the phase transition of relaxation can also be described by another empirical parameter such as Δ*T*_diffuse_ defined as:^58^10
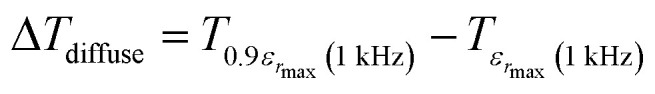
where 
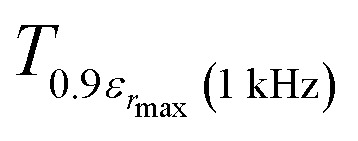
 corresponding to 90% of the temperature of the maximum of the real dielectric permittivity *ε*_*r*_max__ and 
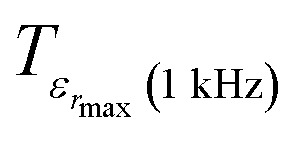
 is their associated maximum at 1 kHz.

In order to quantify the frequency dispersion in the dielectric behavior, we define a parameter 
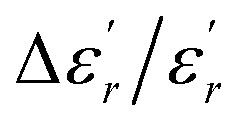
as:11
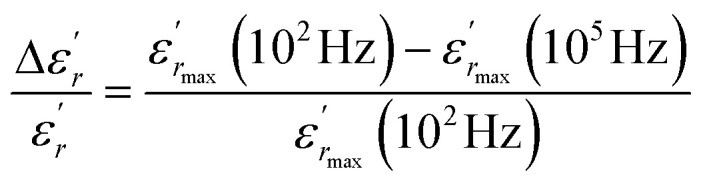


The values of Δ*T*_diffuse_ and 
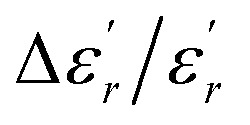
 calculated and summarized in Table 2.

To further understand the relaxor nature of our compound, three models were used to describe the evolution of the maximum peak temperature of permittivity depending on frequency. The first model is that of Debye model based upon classical dielectric media. In Debye relaxation, dipoles are free to rotate and are thermally activated. The dipoles moments have the same values and there is no interaction between the dipoles; which can be written as follows:^59^12
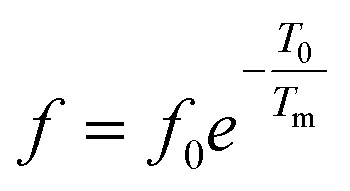
where *f* is the attempt frequency, *T*_0_ is the ratio of the activation energy to the Boltzmann constant *k*_B_, *T*_m_ is the temperature corresponding to this maximum.

To better understand relaxor ferroelectric character, the maximum dielectric constant (*T*_m_) is found to obey Vogel–Fulcher (V–F) model.^60^ This model takes into account the interactions between the dipoles. These interactions make the dipoles freeze at a particular temperature called freezing temperature. This model, described by the relation similar to that recognized for glasses,^61^ can be expressed as follows:13
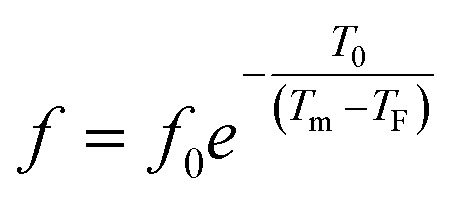
where *T*_0_ is the ratio of the activation energy to the Boltzmann constant *k*_B_. *f*, *f*_0_ and *T*_F_, are respectively, the measurement frequency, the attempt frequency of dipole reorientation and the freezing temperature of the polarization below which the dynamic reorientation of the dipolar cluster polarization is not thermally activated.

**Fig. 7:**
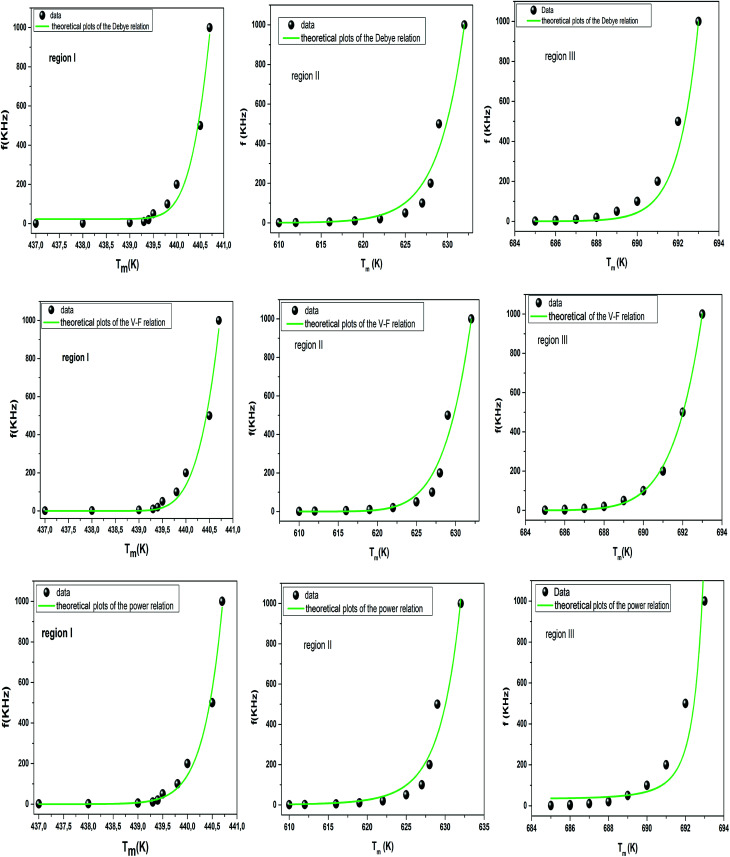
Fitting of Debye, Vogel–Fulcher and Power models to the dielectric relaxation data of the Ba_0.97_Bi_0.02_Ti_0.9_Zr_0.05_Nb_0.04_O_3_ ceramic.

The experimental values are fitted to eqn (12) and (13), and are shown in Fig. 7. The fitting results were computed for the sample and are given in Table 3, for *T*_F_ = 586 K and *E*_a_ = 0.052 eV for region II and *T*_F_ = 669 K and *E*_a_ = 0.033 eV for region III. Similarly, the low values of the activation energy are also found by Zhang *et al.*^62^ for 0.9Pb(Mg_1/3_Nb_2/3_)O_3_-0.1PbTiO_3_ relaxors (*E*_a_ ≈ 0.012 eV), F. Bourgiba *et al.*^63^ for BaTi_0.5_(Fe_0.33_W_0.17_)O_3_ ceramics (*E*_a_ ≈ 0.0494–0.0586 eV) and Tinberg^64^ (1 − *x*)BaTiO_3_–*x*BiScO_3_ films (*E*_a_ ≈ 0.05–0.12 eV). The activation energy *E*_a_, much lower in our ceramic, reflected a lower barrier between two potential wells. This range of features relating to the potential well reflected different polarization mechanisms. The parameters (*E*_a_, *T*_F_) indicate that the relaxation behavior is triggered by monopolars and similar to the thermally activated process of the spin glass in which the freezing process is controlled by cluster flipping and the intercluster interaction mechanism.

To overcome this limitation of V–F model, Cheng *et al.*^65^ proposed a power law:14
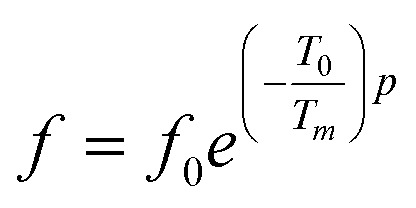
where *T*_0_ is the ratio of the activation energy to the Boltzmann constant *k*_B_. The value of the parameter *p* is a constant associated with the degree of relaxation of the material and can be used to describe the dielectric relaxation strength (DRS). A system exhibits a stronger dielectric relaxation for smaller value of *p*. If the value of *p* = 1, the power model reduces to Debye model and will have strongest dielectric relaxation. For very low value of *p* (=∞), the system behaves like normal ferroelectrics and do not show any relaxation phenomena.

The fitted curves for Power model are shown in Fig. 7. The values obtained for their parameters are listed in Table 3. We notice that parameter *p* (Table 4) is in agreement with those of the diffuseness coefficient *γ* and Δ*T*_m_, which confirms the relaxor behavior of the BBTZN sample.^66^ The pre-exponent factor *f*_0_ provides an idea about the size of polar clusters and the degree of interaction between them. Smaller the size of the cluster, lower the interaction between them and hence the larger value of *f*_0_ and *vice versa*. The value of *p* determines the degree of relaxation and hence the rate of growth of polar clusters. The higher the relaxation is, the slower the rate of growth of polar clusters is. The observed relaxation is related to the slower rate of growth polar clusters. In this compound the Polar clusters have undergone three relaxation areas which can increase their size and this may be a source of the relatively high grain size observed by SEM.

**Table:** Values of factors: Ea the thermal activation energy, the limit frequency of resonance of dipoles at high temperature *f*_0_ and the freezing temperature *T*_F_ of the dipoles for the Ba_0.97_Bi_0.02_Ti_0.9_Zr_0.05_Nb_0.04_O_3_ ceramic

Ba_0.97_Bi_0.02_Ti_0.9_Zr_0.05_Nb_0.04_O_3_		*f*_0_ (Hz)	*E*_a_ (eV)	*T*_F_ (K)	*p*	χ^2^
Debye model	Region I	1.186 × 10^78^	5.21	—	—	0.9745
	Region II	4.175 × 10^66^	2.8	—	—	0.95583
	Region III	3.35 × 10^68^	4.23	—	—	0.96217
Vogel Fulcher model	Region I	4.08 × 10^6^	0.02	437	—	0.97969
	Region II	6.1 × 10^8^	0.052	586	—	0.99862
	Region III	1.03 × 10^10^	0.033	669	—	0.96206
Power model	Region I	6.45 × 10^13^	0.05	—	16.52	0.98675
	Region II	2.44 × 10^4^	0.011	—	9.89	0.95224
	Region III	2.6 × 10^5^	0.021	—	7	0.97441

Comparing χ^2^ values, we notice the good agreement of the data with Vogel–Fulcher relationship, which suggests that the relaxor behavior in our compound is analogous to that of dipolar glass with a polarization fluctuation above a static freezing temperature.

**Fig. 8:**
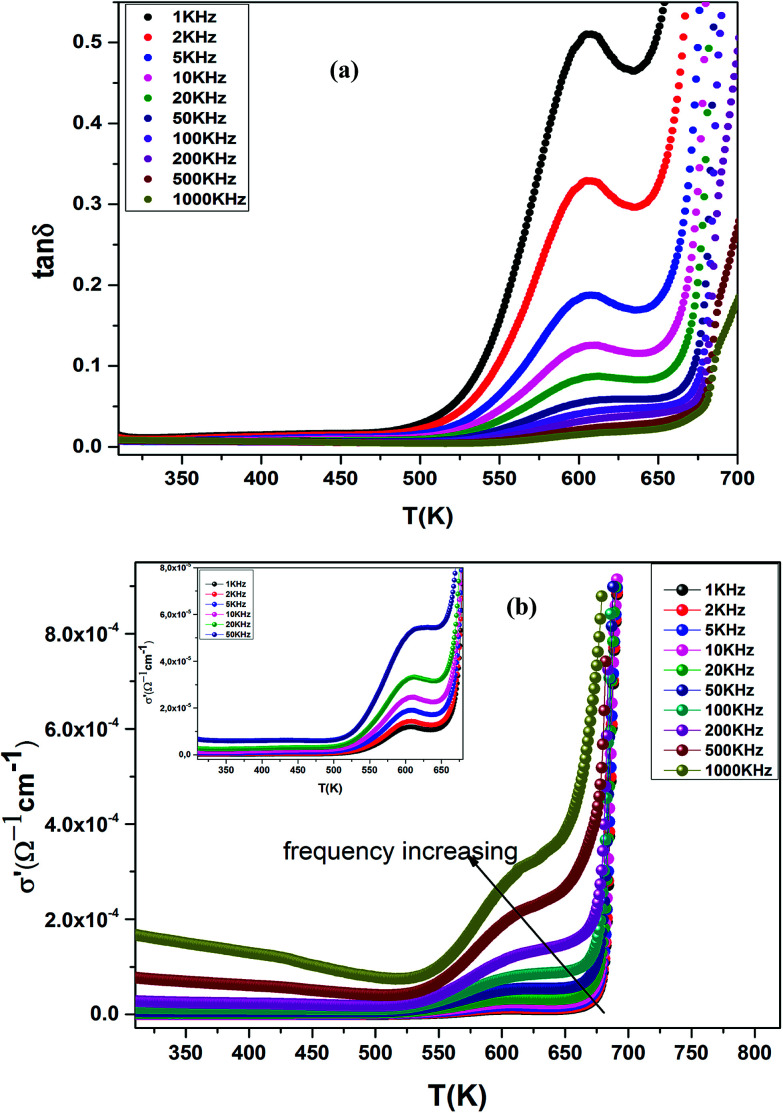
(a) Temperature and frequency dependence of the dielectric loss (tan *δ*) for Ba_0.97_Bi_0.02_Ti_0.9_Zr_0.05_Nb_0.04_O_3_, (b) the dielectric conductivity of this compound.

The dielectric loss (tan *δ*) of the compound BBTZN as a function of temperature up to 700 K at various frequency ranges from 1 kHz to 1 MHz is represented in Fig. 8(a). We observe a marked peak that shifts to higher frequencies with temperature, resembling the relaxor type dielectric behavior. The appearance of a broadened peak in the dielectric loss as a function of temperature is indicative of a transition from one type of relaxation process to another taking place at a characteristic temperature and may be attributed to the combined effect of the reorientation of thermally activated polar flips and loss of polarization with the rise in temperature.^67^ In addition, the high dielectric loss at higher temperature is supposed to be induced by the thermally activated space charge contribution (Maxwell–Wagner type).^68^

The electrical conductivity (*σ*′) of a dielectric material can be presented in terms of the dielectric loss (tan *δ*) by the relation:^69^15
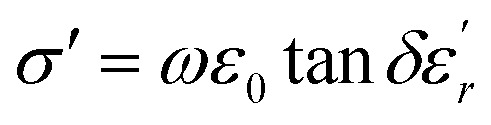
where *ω* = 2π*f*, *f* is frequency, *ε*_0_ and *ε*_*r*_ are the permittivity of free space and dielectric constant, respectively, and tan *δ* is dielectric loss.

The temperature dependence of the electrical conductivity (*σ*′) for BBTZN is presented in Fig. 8(b). Results shown that *σ*′ increases simultaneously with increasing the temperature and frequency. Moreover, the electrical conductivity curves show a typical feature of relaxation behavior. These results are in agreement with those obtained in the dielectric loss (tan *δ*) (Fig. 8(a)).

The complex electric modulus was calculated from the dielectric permittivity [ε*(*ω*)] using the following relation:^70^16
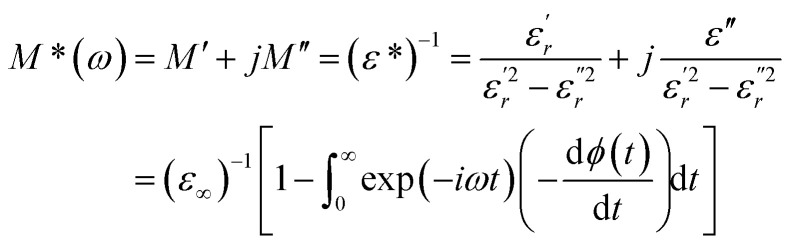


With17
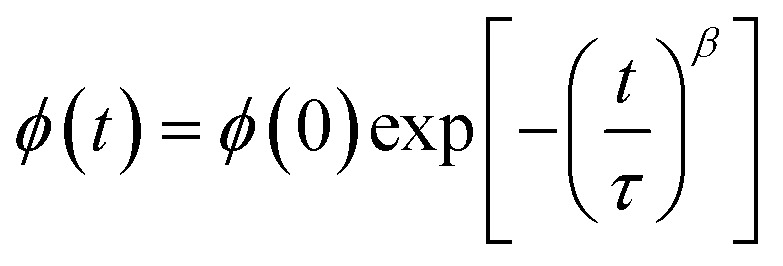
where 
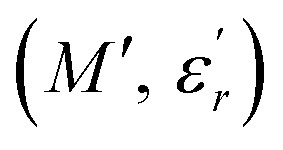
 and 
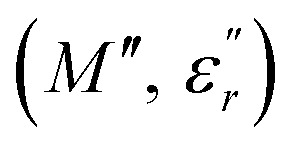
 are the real and imaginary components of modulus and permittivity, respectively, 
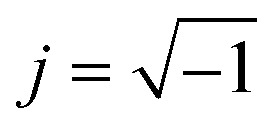
 is the imaginary factor, *ε*_∞_ is the asymptotic value of real part of the dielectric constant, *Φ*(*t*) is the stretched exponential function of a material and *β* (0 < *β* < 1) is the stretching coefficient and gives the degree of correlation between ions in ionic transport corresponding to complete uncorrelated ionic motion when its value is unity.

**Fig. 9:**
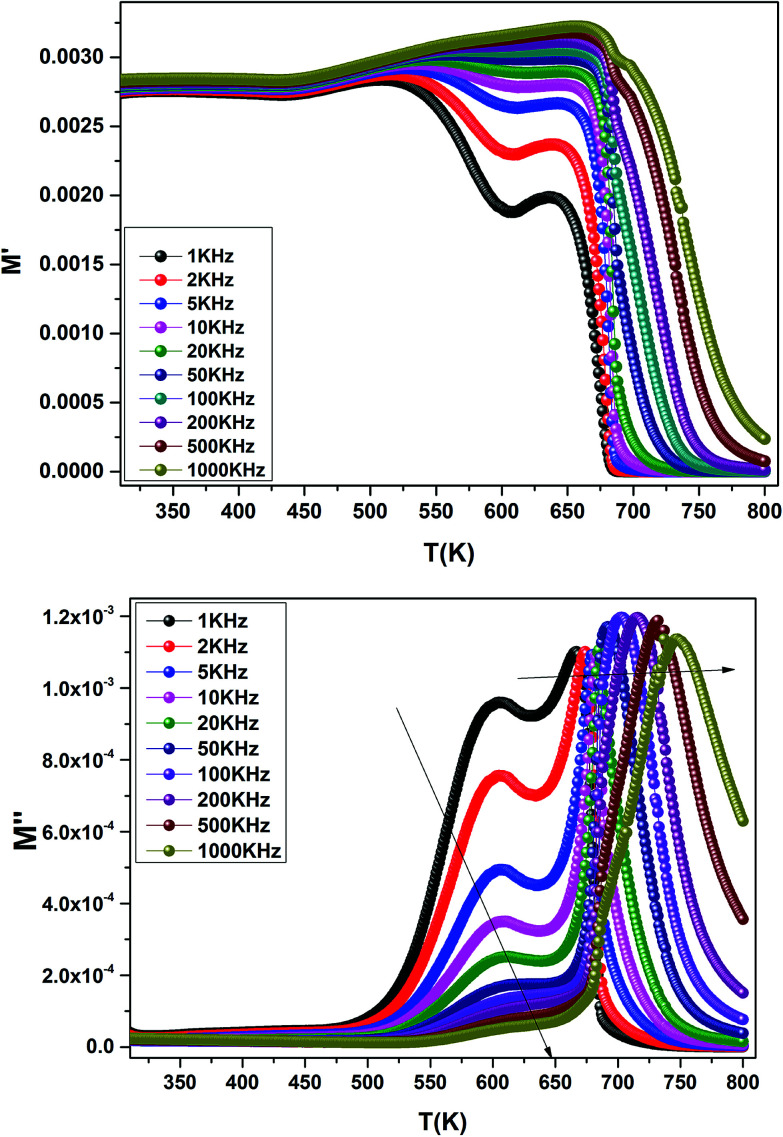
Temperature dependence of the electric modulus (M′ and M′′) for Ba_0.97_Bi_0.02_Ti_0.9_Zr_0.05_Nb_0.04_O_3_ at various frequencies.

The temperature dependence of the electric modulus *M*′ and *M*′′ for various frequencies is shown in Fig. 9(a and b) for Ba_0.97_Bi_0.02_Ti_0.9_Zr_0.05_Nb_0.04_O_3_. We observed that, at lower temperature, *M*′ tends to a constant value for all frequencies. This dependence indicates that the dielectric constant of the sample is thermally activated, similar to that of the dielectric constant spectrum by the presence of the phase transition with relaxor behavior (Fig. 3).

In addition, we see a two characteristic peaks in the temperature range. One is in the range of 300 to 600 K and the other lies between 600 and 800 K. Correspondingly, *M*′′ shows two peaks in the same temperature ranges. Both peaks shift to high temperature with increasing frequency indicating a thermally activated relaxation. This peaks were ascribed to the Maxwell–Wagner (M–W) space charge relaxation phenomenon, such as space charges, charged defects, and related defect complexes.^71^ We attribute the low temperature peak to the dielectric relaxation and the high-temperature peak to the conduction process, similar to that of the conductivity spectrum (Fig. 8(b)). A thermally activated conduction process of defects produced during high temperature sintering such as oxygen vacancies and the presence of small grain boundary. Electrical inhomogeneities could be the possible reason for these high temperature dielectric relaxations.^72^

The results obtained in this study indicate that our ceramics are of extreme significance as far as their technological and industrial applications are concerned.

## 4. Conclusion

In summary, we have synthesized a new ceramic compound of composition Ba_0.97_Bi_0.02_Ti_0.9_Zr_0.05_Nb_0.04_O_3_ by molten-salt method. Rietveld analysis indicated that this sample crystallizes in pseudocubic structure with a *Pm*3̄*m* space group. The temperature dependence of the dielectric properties was investigated in the frequency range 1 kHz to 1 MHz. Importantly, Bi^3+^ substitution in A site and (Zr^4+^, Nb^5+^) for Ti^4+^ in B site is found to induce chemical and structural inhomogeneity that leads lead to the formation of polar nanoregions, local structure distortions, and local charge imbalance reflected as increasing diffuseness in the transition. Indeed, three regions of dielectric relaxations were observed in the title perovskite at the temperature ranges of 350–500 K, 500–620 K and 620–720 K with a maximum in the dielectric permittivity that shifted to a higher temperature with increasing frequency. Moreover, to investigate the degree of the DPT, the modified Curie–Weiss law has been introduced to describe the relaxation behavior attributed to the contribution of PNRs. Using Vogel–Fulcher (V–F) model, the estimated value of parameters (*E*_a_, *T*_F_) indicate a low barrier between two potential yield to the presence of different polarization mechanisms in this compound. These observations suggest that the present system can be considered as a potential lead-free material for the nanotechnologies applications.

## Conflicts of interest

There are no conflicts to declare.

## Supplementary Material
